# Abdominal compartment syndrome and ruptured aortic aneurysm: Validation of a predictive test (SCA-AAR)

**DOI:** 10.1097/MD.0000000000011066

**Published:** 2018-06-22

**Authors:** Betty Leclerc, Lucie Salomon Du Mont, Anne-Laure Parmentier, Guillaume Besch, Simon Rinckenbach

**Affiliations:** a Vascular Surgery Unit, University Hospital of Besançon; b Department of Anesthesiology and Intensive Care Medicine, University Hospital of Besançon; c EA, University of Franche-Comté, Besançon; d UMR Chrono-Environnement, University of Franche-Comté, La Bouloie-UFR Sciences et Techniques, Besançon Cedex; e Clinical Methodology Center, University Hospital of Besançon, 2 place Saint Jacques, 25030 Besançon, France.

**Keywords:** abdominal compartment syndrome, decompressive laparotomy, ruptured aortic aneurysm

## Abstract

**Background:**

The abdominal compartment syndrome (ACS) has been clearly identified as being one of the main causes of mortality after ruptured abdominal aortic aneurysm (rAAA). The ACS is defined as a sustained intra-abdominal pressure > 20 mm Hg associated with a new organ dysfunction or failure. A pilot study was conducted and found that the threshold of 3 among 8 selected criteria, we would predict an ACS occurrence with a 54% positive predictive value and a 92% negative predictive value. But a multicentric prospective study was clearly needed to confirm these results. The outcome of this new study is to assess the qualities of a predictive test on occurrence of the ACS after rAAA surgery.

**Methods:**

This is a 30 months prospective cohort study conducted in 12 centers and 165 patients will be included. All patients with a rAAA will be consecutively included, whatever the surgical treatment. At the end of surgery, all patients have an abdominal closure and a monitoring of intrabladder pressure will be established every 3 to 4 hours. Decompressive laparotomy will be indicated when ACS occurs. Follow-up period is 1 month. Eight pre- and per-operative criteria will be studied: anemia, hypotension, cardiac arrest, obesity, massive fluid resuscitation, transfusion, hypothermia, and acidosis.

**Discussion:**

In the literature, there is no recommendation about prophylactic decompression, but early decompressive laparotomy appears to improve survival. This study should make it possible to establish a predictive test, detect the ACS early, and consider a prophylactic decompression in the operating room.

**Trial registration:**

ClinicalTrials.gov, NCT02859662, Registered on 4 August 2016.

## 1 Background

Despite improved management techniques of ruptured abdominal aortic aneurysm (rAAA), especially in vascular surgery, the perioperative mortality rate has not decreased these last years. The preoperative deaths are mainly due to uncontrolled bleeding and its consequences whereas the postoperative deaths are rather related to multiple organ failure that could be explained by a misdiagnosis or a delayed diagnosis of an occurring abdominal compartment syndrome (ACS).

The ACS has been clearly identified as being one of the main causes of mortality after rAAA.^[1,2]^ The ACS is defined as a sustained intra-abdominal pressure greater than 20 mm Hg associated with a new organ dysfunction or failure.^[3–7]^ Mainly risk factors known for intra-abdominal hypertension (IAH) (intra-abdominal pressure > 12 mm Hg) are diminished abdominal wall compliance, increased intra-abdominal contents, capillary leak or fluid resuscitation, others than coagulopathy, obesity, sepsis, shock, and hypotension.^[3]^

The mortality rate of ACS can reach 80% to 100% in the absence of treatment.^[8]^ Early detection and appropriate management of intra-abdominal hypertension and ACS have reduced their mortality.^[1]^ In addition to the medical management, decompressive laparotomy with temporary abdominal closure is the treatment of ACS.^[8–11]^ Importance of screening as well as early management has been demonstrated, but decompressive laparotomy has its own complications such as wall pain, evisceration or increase of infection, and could not be propose systematically.^[10,12–20]^

The identification of patients who are at high risk of ACS after surgical repair of rAAA in the operating room could allow detecting and managing earlier patients with an increase in intra-abdominal pressure and ACS. Several factors related to the occurrence of intra-abdominal hypertension and ACS has been identified in the literature.^[12,16,19]^ Ruptured abdominal aneurysm is one of the risk factors. Among the others risk factors identified during the last consensus conference, we selected 6 that could be diagnosed in the pre- and intraoperative period: systolic blood pressure, body mass index, massive fluid resuscitation, massive transfusion, hypothermia, and acidosis.^[3,7]^ We selected 2 additional criteria, anemia and cardiac arrest, more specific to rAAA. These 8 criteria are easily collected during the initial management, correspond to data of the usual management in these patients and easy to access even in the operating room. We chose to analyze both surgical techniques (open or endovascular) together because the prevalence of ACS in the literature is the same.^[10,12–17,21,22]^

Previously, we have conducted a retrospective pilot study in our center, which found that with the threshold of 3 among 8 selected pre- and intraoperative criteria, we would predict an ACS occurrence with a 54% positive predictive value and a 92% negative predictive value. The prevalence of ACS in our study was 17% and the mortality rate in the group of patients with an ACS was 37.5%.^[23]^ Nevertheless, it was a retrospective, monocentric study with a small population without systematically measure of intra-abdominal pressure and with posteriori diagnosis of ACS. A multicentric prospective study was clearly needed to confirm these results.

This study aims to specify a predictive test for the occurrence of an ACS after rAAA surgery. Early detection of the ACS could enable rapid management of patients and influence the mortality rate.

## 2 Methods

### 2.1 Study design

This is a French prospective cohort study on patients presented a rAAA. The number of patients to be included is 165. All patients with a rAAA will be consecutively included, whatever the surgical treatment, open surgery or endovascular surgery. The estimated duration of recruitment is 30 months.

The primary endpoint of the study is the occurrence of an ACS within 5 days after surgery. The predictive capacity of the occurrence of ACS will be measured by sensitivity, specificity, positive and negative likelihood ratio, positive and negative predictive value.

The secondary endpoints are the occurrence of an ACS within 5 days after surgery according to the type of surgical technique (open surgery or endovascular surgery), death within 30 days of intervention, and hospital stay (intensive care unit stay and total hospital stay).

The 8 pre- and per-operative criteria are:

(1) Anemia with hemoglobin < 10 g/dL
(2) Persistent of a systolic blood pressure lower than 90 mm Hg
(3) Cardiac arrest
(4) Body mass index (BMI) > 30
(5) Massive fluid resuscitation of more than 3500 mL/h for at least one hour
(6) Transfusion ≥ 10 units of red blood cells since the beginning of the treatment
(7) Hypothermia with a temperature of 33°C or below
(8) Acidosis with a pH ≤ 7.2.

When a patient with an rAAA arrives at the emergency room, he can be included in the protocol. According to its clinical condition, information on the study will be given to him and nonobjection consent. If the patient is unable to give consent, his or her family will be contacted to confirm non opposition to data collection. The data collected are usual and the management of rAAA is the same. No further examination is necessary for the study because it is an observational prospective study.

At admission, the following data will be collected: age, gender, weight, height, BMI, medical, and surgical history. During the pre- and intraoperative period: pre- and perioperative criteria, abdominal closure conditions, type of intervention, surgical procedure, type of anesthesia, clamping time, blood loss, and anesthetic conditions.

**Figure 1:**
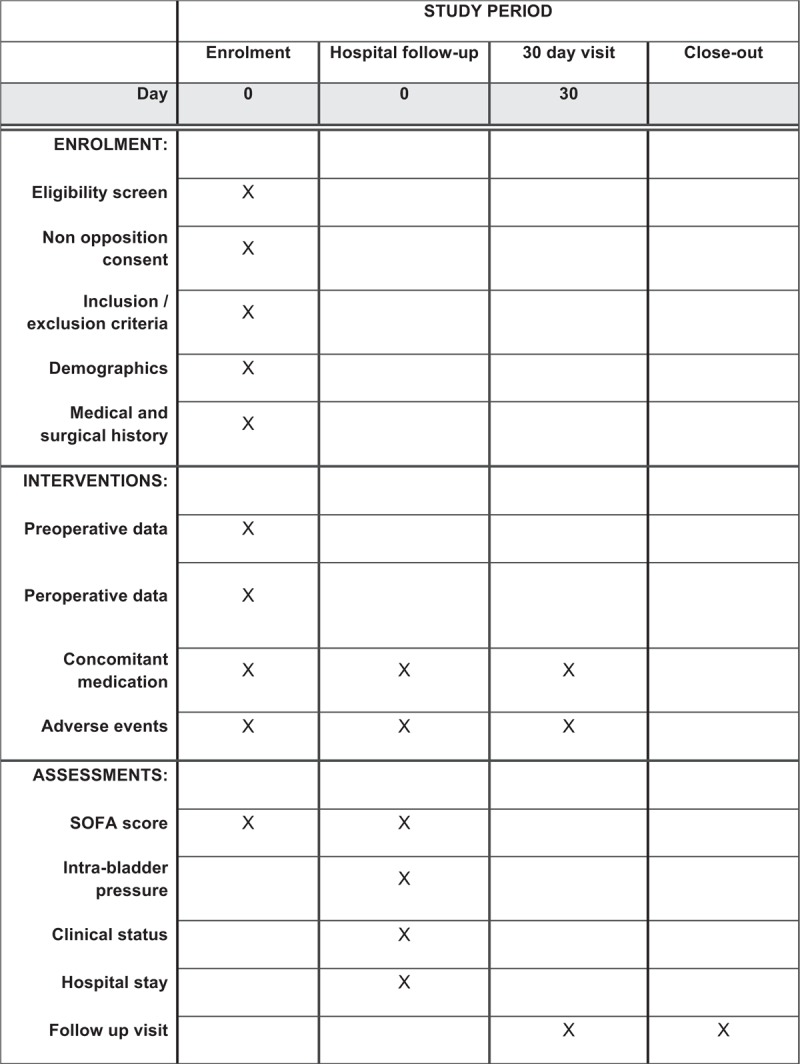
Schedule of enrolment, interventions and assessments.

At the end of surgery, all patients have an abdominal closure. Patients are transferred in intensive care unit for clinical and paraclinical monitoring. Normal intra-abdominal pressure, range between 0 to 5 mm Hg, is usually measured by intra bladder pressure and it is a reproducible and non-invasive method.^[21,24–26]^ So during the first days, monitoring of intra bladder pressure will be established. The measurements will be carried out every 3 to 4 hours according to the centers, during the critical clinical situation and/or if the results are greater than 12 mm Hg. The end of the critical period is defined by the cessation of sedation. If clinical worsening occurs, intra bladder pressure will be monitored and repeated every 3 to 4 hours again (Fig. 1). Decompressive laparotomy will be indicated when bladder pressure is > 20 mm Hg associated with a new organ failure. However, isolated elevation of bladder pressure could perform decompression laparotomy. Management of IAH and ACS will be done according to the usual practice and current recommendations.^[3–5,11,14]^ All patients will be monitored over a period of 30 days.

During the postoperative period, the following data will be collected: APACHE II and SOFA score,^[27,28]^ clinical and paraclinical follow-up, intra bladder pressure, hemodynamic stability, therapeutic used to control or decrease intra-abdominal pressure, complications (type, chronology, treatment), additional surgical procedure, secondary decompression laparotomy, condition of closure of the abdomen in case of decompression laparotomy.

**Figure 2:**
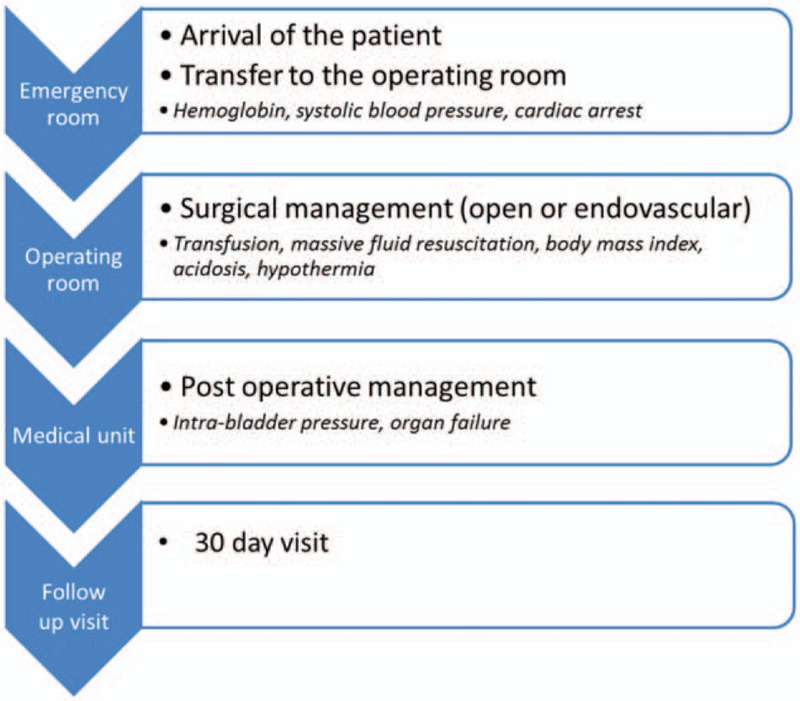
Study design.

At 1 month, follow-up visit and clinical examination is done. These data will be collected: 30 days mortality, intensive care stay, total hospital stay, cause of death, and clinical examination (Fig. 2).

A committee will be necessary for the a posteriori validation of the ACS diagnosis. It will consist of 2 persons (a surgeon and an intensivist) who are not aware of the patient's identity, surgeon's identity, and the value of the 8 criteria. For each patient, the data used to evaluate the diagnosis will be collected in a standardized file and presented to the member of the validation committee during specific sessions.

### 2.2 Objectives

The primary objective of the study is to assess the qualities of a predictive test on the occurrence of this syndrome after surgery of rAAA.

Secondary outcomes were to compare the test between surgical techniques (endovascular or open), 30 days mortality rate and the duration of hospitalization (intensive care unit stay and total hospital stay).

### 2.3 Inclusion and exclusion criteria

Male or female patients to be enrolled in the study should meet the following inclusion criteria: age ≥ 18 years, to be hospitalized in participating hospitals, rAAA confirmed and operated.

Exclusion criteria are pregnancy, patients with bladder tumor or bladder surgery or trauma bladder can distort bladder pressure measurement, cystectomy, patients died before arrival in the operating room, patients died during surgery or within one hour of the initial surgical procedure, and patients whose abdominal closure at the end of the procedure is not possible.

### 2.4 Data management

Data will be managed using the Clean Web business application, an electronic clinical trial management solution from Telemedicine Technology. This solution meets the requirements of BPC, ICH, 21 CFR Part 11 (FDA) control and ensures secure data hosting. It also allows for a thorough implementation of users’ access rights based on their profile on the study.

### 2.5 Ethical conduct of the study and informed consent

This study will be conducted in 9 centers in France: members of the French Association of Vascular Research (Association Universitaire de Recherche Clinique): Besançon, Bordeaux, Clermont-Ferrand, Dijon, Lyon, Nancy, Nantes, Rennes, and Strasbourg, and 3 others centers: Colmar, Mulhouse and Belfort (Nord Franche-Comté Hospital). Informed will be systematically provided to the patients or their families.

### 2.6 Statistical analysis

The quantitative variables will be described using the mean and standard deviation, the median, and the minimum and maximum values. The qualitative variables will be described by the proportion and the percentage of each class.

As previously demonstrated, the test will be considered positive if the patient presents at least 3 criteria on the 8.^[23]^ Specificity, sensitivity, positive, and negative likelihood ratios, positive and negative predictive values with their confidence interval will be calculated for the new predictive test.

The comparison between surgical techniques used will be carried out with a Chi^2^ test or an exact Fisher test. A logistic regression carried out on sensitivity analysis will be done to build a predictive score and confirm the result.

### 2.7 Sample size calculation

For a sensitivity of 0.75, a precision of 0.15 and an alpha risk of 5%, the number of subjects required is 33 patients with ACS. For a prevalence of ACS at 20%, the number of patients required is 165.^[10,12–19,21,22]^

## 3 Discussion

The estimated recruitment is 30 months and the number of patients to be included is 165. The recruitment will be consecutive and with no patient selection profile. All patients with a rAAA will be consecutively included in each center.

Inclusion will initially begin in our region and then extend to nonregional centers. This progressive opening of the centers will ensure the correct inclusion of patients. The principal investigator will visit each center to explain the criteria measurement procedures and the intra bladder pressure measurement to ensure homogenous management.

Decompressive laparotomy is the only treatment for ACS and there is no recommendation about the prophylactic management of the ACS. In the literature, we found that early decompressive laparotomy appears to improve survival than late decompression.^[7]^

A prophylactic decompression could be proposed to avoid ACS after rAAA surgery, and prevent occurrence of complications. But, there are complications after an open abdomen, especially for a long time, such as cutaneous complications, enterocutaneous fistula, intra-abdominal infection or evisceration, so it would necessary to early detect the ACS and identify patients at risk to develop an ACS, and do not propose systematic laparostomy at the end of surgery.

Later, the aim of the future study will be to screen ACS and manage patient as soon as possible to avoid it. We would like to carry out a prospective randomized study between early laparostomy versus delayed laparostomy to compare 30 days mortality rate.

## Acknowledgments

We would like to thank the participants in the study of all centers: Dr Besch, Pr Midy, Dr Petit, Pr Rosset, Dr Gillart, Pr Steinmetz, Dr Anciaux, Pr Lermusiaux, Dr Floccard, Dr Settembre, Dr Guerci, Pr Goueffic, Dr Asehoune, Dr Rozec, Pr Chakfe, Dr Collange, Dr Cardon, Dr Seguin, Dr Kretz, Dr Gaertner, Dr Ghassani, Dr Real, Dr Leroux, Dr Badie, Dr Malfroy, and Dr Kalakhy.

## Author contributions

SR, BL, and LSDM conceived the study. GB and ALP contributed to the study design. BL, LSDM, and SR drafted the manuscript. All authors have read and approved the final manuscript.

**Conceptualization:** Betty Leclerc, Lucie Salomon Du Mont, Simon Rinckenbach.

**Data curation:** Betty Leclerc.

**Formal analysis:** Anne-Laure Parmentier.

**Investigation:** Betty Leclerc.

**Methodology:** Anne-Laure Parmentier.

**Supervision:** Lucie Salomon Du Mont, Anne-Laure Parmentier, Simon Rinckenbach.

**Validation:** Simon Rinckenbach.

**Visualization:** Guillaume Besch.

**Writing – original draft:** Betty Leclerc.

**Writing – review & editing:** Betty Leclerc, Lucie Salomon Du Mont, Simon Rinckenbach.
